# Brain multi-contrast, multi-atlas segmentation of diffusion tensor imaging and ensemble learning automatically diagnose late-life depression

**DOI:** 10.1038/s41598-023-49935-z

**Published:** 2023-12-20

**Authors:** Kostas Siarkos, Efstratios Karavasilis, Georgios Velonakis, Charalabos Papageorgiou, Nikolaos Smyrnis, Nikolaos Kelekis, Antonios Politis

**Affiliations:** 1 Division of Geriatric Psychiatry, First Department of Psychiatry, National and Kapodistrian University of Athens, Athens, Greece; 2 Medical School, Democritus University of Thrace, Alexandroupolis, Greece; 3 Second Department of Radiology, Attikon General University Hospital, School of Medicine, National and Kapodistrian University of Athens, Athens, Greece; 4 University Mental Health, Neurosciences and Precision Medicine Research Institute “Costas Stefanis”, Athens, Greece; 5 Second Department of Psychiatry, Attikon General University Hospital, School of Medicine, National and Kapodistrian University of Athens, Athens, Greece; 6 Department of Psychiatry, Division of Geriatric Psychiatry and Neuropsychiatry, Johns Hopkins Medical School, Baltimore, USA

**Keywords:** Bioinformatics, Medical research

## Abstract

We investigated the potential of machine learning for diagnostic classification in late-life major depression based on an advanced whole brain white matter segmentation framework. Twenty-six late-life depression and 12 never depressed individuals aged > 55 years, matched for age, MMSE, and education underwent brain diffusion tensor imaging and a multi-contrast, multi-atlas segmentation in MRIcloud. Fractional anisotropy volume, mean fractional anisotropy, trace, axial and radial diffusivity (RD) extracted from 146 white matter parcels for each subject were used to train and test the AdaBoost classifier using stratified 12-fold cross validation. Performance was evaluated using various measures. The statistical power of the classifier was assessed using label permutation test. Statistical analysis did not yield significant differences in DTI measures between the groups. The classifier achieved a balanced accuracy of 71% and an Area Under the Receiver Operator Characteristic Curve (ROC-AUC) of 0.81 by trace, and a balanced accuracy of 70% and a ROC-AUC of 0.80 by RD, in limbic, cortico-basal ganglia-thalamo-cortical loop, brainstem, external and internal capsules, callosal and cerebellar structures. Both indices shared important structures for classification, while fornix was the most important structure for classification by both indices. The classifier proved statistically significant, as trace and RD ROC-AUC scores after permutation were lower than those obtained with the actual data (P = 0.022 and P = 0.024, respectively). Similar results were obtained with the Gradient Boosting classifier, whereas the RBF-kernel Support Vector Machine with k-best feature selection did not exceed the chance level. Finally, AdaBoost significantly predicted the class using all features together. Limitations are discussed. The results encourage further investigation of the implemented methods for computer aided diagnostics and anatomically informed therapeutics.

## Introduction

While depression and related symptoms are a common mental health problem in older people, late-life depression (LLD) is underdiagnosed and undertreated^1^ and has been associated with cognitive deterioration and dementia^2,3^. Brain structural changes in LLD have been observed with magnetic resonance imaging (MRI)^4^ and histology^5,6^. Regarding white matter (WM) changes, diffusion weighted imaging (DWI) and its main application, diffusion tensor imaging (DTI) has revealed significant alterations in patients with LLD, compared to non-depressed healthy controls^7,8^ and these WM changes may precede the onset of depression^9^. However, variability in the results exists^10–12^ while distinct neuroanatomical dimensions based on MRI have been identified in LLD when large scale data are analyzed^8^. Therefore, it is important to characterize and better understand the white matter alterations in LLD, in order to assist with correct diagnosis and development of targeted and more personalized treatments.

Machine learning (ML) is receiving a growing interest in neuroimaging literature and is continuously used for classification purposes in a variety of conditions including developmental, neurocognitive and psychiatric disorders^13^. However, studies on ML methods applied to neuroimaging in LLD are sparse and have utilized T1^14^, functional MRI (fMRI)^15^ and multimodal MRI^8,16,17^. While image segmentation is a key step in brain imaging analysis, segmentation of the WM based on multiple DTI contrasts and atlases has never been reported in LLD, to the best of our knowledge.

In this study, we aimed to assess WM changes in LLD using a framework for DTI segmentation not previously used in this population. We then aimed to develop a ML model based on the segmentation output, to automatically diagnose LLD and never depressed individuals. The discrimination performance of the model was evaluated with a variety of measures and the statistical power of the classifier was tested.

## Results

The demographic and clinical characteristics of patients with LLD and HC are shown in Table 1.

**Table 1:** Demographic and clinical variables.

	LLD group (N = 26)	NC group (N = 12)	*F*-statistic	*p*-value
Age (years, mean ± SD)	68.38 ± 8.48	66.58 ± 4.60	0.474	0.496
Gender (male:female)	11:15	9:3	NA^b^	0.086^a^
Education (years, mean ± SD)	12.50 ± 3.37	14.08 ± 2.19	2.196	0.147
MMSE score	29.00 ± 0.85	29.42 ± 0.51	2.453	0.126
GDS score	11.88 ± 1.18	1.42 ± 1.08	680.862	5.6 × 10^−25^

All* p*-values were obtained from a between group one-way Analysis of Variance test, unless otherwise specified.

*LLD* late-life depression, *NC* normal control, *SD* standard deviation, *MMSE* mini-mental state examination, *NA* not applied, *GDS* Geriatric Depression Scale.

^a^Fisher's exact test. A *p*-value of < 0.05 denotes no relationship between gender and group, expressed as the sum of probabilities obtained from a permutation procedure of all the gender x group contingency tables less likely than or equal likely to the observed table.

^b^Test statistic value is not reported, as the Fisher's exact test performed for gender calculates a sum of frequency probabilities from a permutation procedure to estimate the *p*-value, rather than a test statistic.

### Group differences in DTI

Differences in all DTI measures are presented in Supplementary Table S1. The differences in Fractional Anisotropy (FA) volume were widespread, particularly the fornix, fornix-stria terminalis, internal capsules, left cerebral peduncle, corticospinal tracts, cerebellar regions, superior temporal gyrus, cuneus and the cingulum, while for mean FA, trace, axial diffusivity (AD), and radial diffusivity (RD) the differences were mainly observed in medulla, cerebellum, and midbrain. However, the significant *P*-values from the Mann–Whitney test did not survive after correction for multiple comparisons. Regarding the gender differences between the groups, a correlation analysis was performed to test for an association between predicted class and gender. For each of the 30 classification iterations and DTI metrics, the mean Pearson correlation coefficient (obtained after averaging transformed r to z-values and then transformed back) was r = 0.2, suggesting a weak correlation. Further, to assess for gender bias in the model, we ran the classification selecting gender as the prediction class. We found that DTI features failed to predict the gender. (Supplemental Fig. S4).

### Classification performance and classifier significance

**Figure 1:**
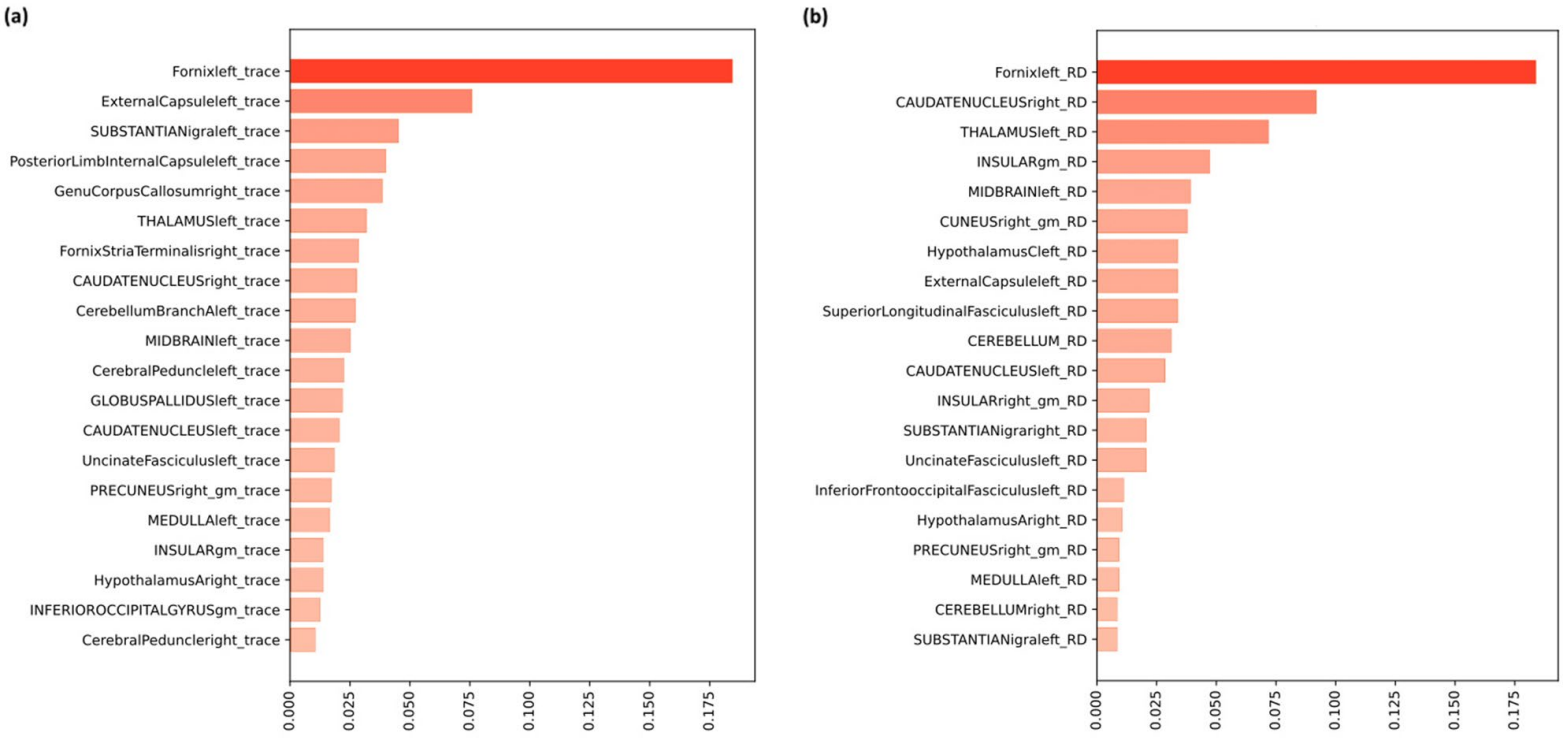
Shown in descending order are the twenty highest trace (**a**) and RD (**b**) relative feature importance determined as the normalized weighted sum of the number of times a given feature is used to split the data in the ensemble. The higher the frequency of a feature being used for splitting, the higher its importance. For example if the fornix has a feature importance of 0.2 it means fornix has a relative importance of 0.2 or a 20% in the ensemble (averaged over 30 repetitions of classification) compared to the other features. *Gm* gray matter.

Classification performance with each WM measure is shown in Table 2 and plotted along with 95% confidence intervals in Supplemental Fig. S2. The classifier successfully discriminated between LLD and NC using trace (balanced accuracy = 71%, ROC-AUC = 0.81) and RD (balanced accuracy = 70%, ROC-AUC = 0.80). The most important discriminative WM regions are shown in Fig. 1. The following regions were important with both indices: the left fornix, right fornix stria terminalis, left thalamus, left substantia nigra, left external capsule, left medulla, left anterior limb of internal capsule, left midbrain, right cuneus, right insular, right caudate nucleus, right and left hypothalamus and cerebellar regions. The corpus callosum, the internal capsule, globus pallidus, and cerebral peduncles were important features only for the classification with trace, while the cuneus and the superior longitudinal fasciculus with RD. Interestingly, fornix was the most important structure for classification with both trace and RD (Fig. 1). Classification using all features as the input revealed a statistically significant model (ROC-AUC = 0.78, p = 0.045 and balanced accuracy = 67%, p = 0.044) (Suppl. Fig. S5) predicting the classes with performances close to Adaptive Boosting (AdaBoost) and Gradient Boosting (GBoost) (Table 2). Similar performances as the AdaBoost were obtained with the Gradient boost classifier (Table 2). Interestingly, the two algorithms shared 12 out of 20 most important features for the classification with both trace (Fig. 1a and Suppl. Fig. S7) and RD (Fig. 1b and Suppl. Fig. S8). The performance of Support Vector Machine (SVM) was low (Table 2).

**Table 2:** Classification performance by the five DTI measures separately and all features together with three algorithms.

DTI index	Classifier	Balanced accuracy (%)	Recall (%)	Precision (%)	F1 (%)	ROC-AUC
FA volume	AdaBoost	55	78	69	71	0.60
	Gboost	47	66	63	62	0.49
	SVM	60	69	74	69	0.68
FA	AdaBoost	50	72	67	67	0.65
	Gboost	46	68	61	63	0.48
	SVM	56	81	72	74	0.52
Trace	AdaBoost	71	84	83	81	0.81
	Gboost	68	88	80	81	0.77
	SVM	57	72	73	70	0.71
AD	AdaBoost	48	72	66	67	0.48
	Gboost	69	88	80	82	0.71
	SVM	52	70	68	66	0.58
RD	AdaBoost	70	86	83	82	0.80
	Gboost	66	85	80	80	0.80
	SVM	57	67	72	67	0.69
All features	AdaBoost	67				0.78

*ROC-AUC* receiver operator characteristic curve-area under the curve, *FA* fractional anisotropy, *AD* axial diffusivity, *RD* radial diffusivity, *AdaBoost* Adaptive Boosting, *GBoost* Gradient Boosting, *SVM* Support Vector Machines.

**Figure 2:**
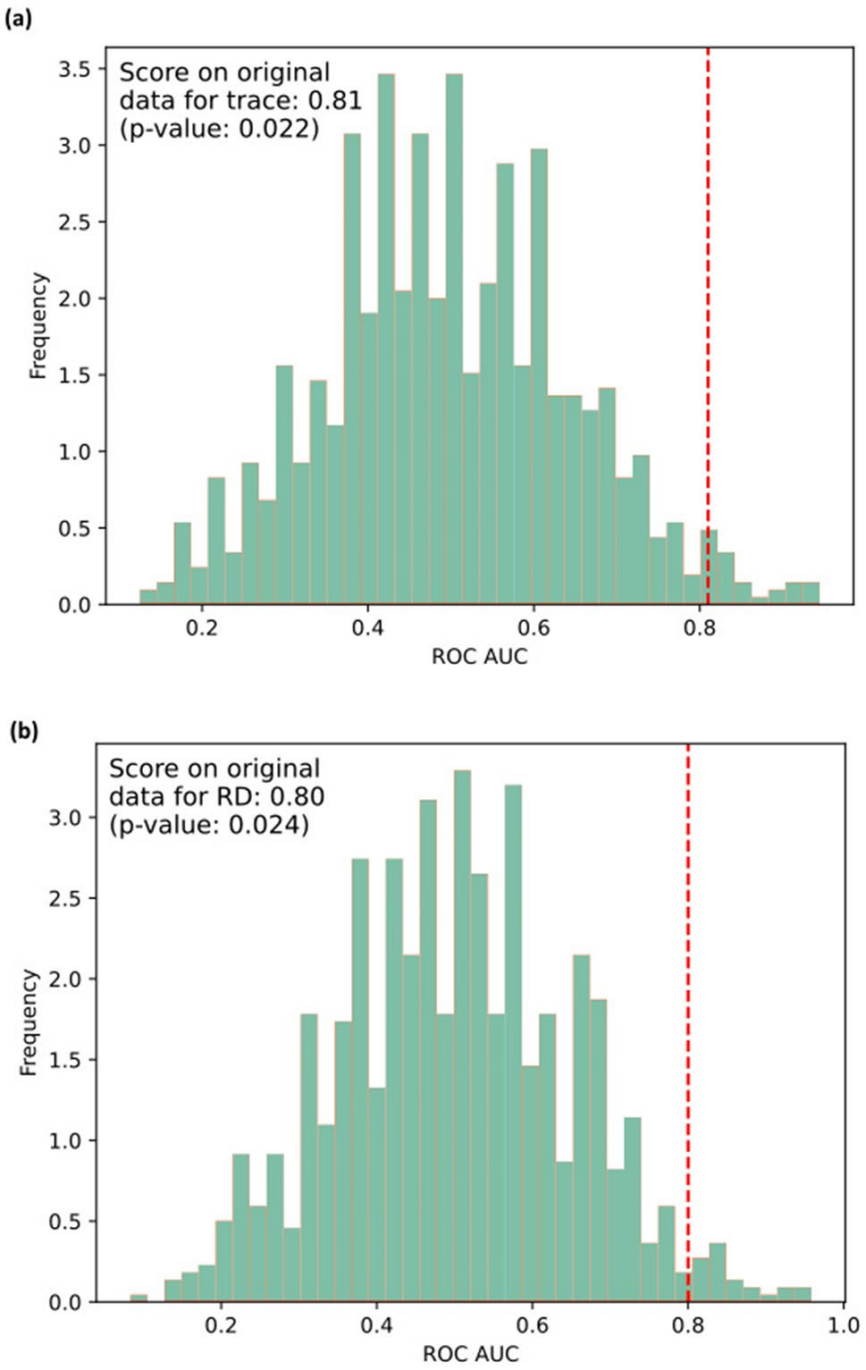
Distribution of ROC-AUC scores (shown in green) obtained with the AdaBoost classifier using cross validation after 1000 label permutations, compared with the score obtained with the actual data (dashed red line) for trace (**a**) and RD (**b**), respectively. Differences are considered significant at the 95% confidence interval level.

In the analysis of classifier’s statistical significance, the ROC-AUC scores obtained with permuted labels were significantly lower than those made with the actual data, using both trace and RD indices (permutation-based P = 0.022 and P = 0.024, respectively) (Fig. 2). Similar results are obtained with balanced accuracy (Fig. S3). This demonstrates that the value of the error in the actual data is small, the prediction accuracy is significantly higher than chance, and the classifier is statistically significant.

## Discussion

In this study, we applied for the first time a multi-contrast, multi-atlas method for automatic DTI segmentation combined with the AdaBoost classifier to classify LLD and HC subjects.

The main findings of our work are: (1) using the trace index, the classifier reached a classification balanced accuracy of 71% and a ROC-AUC of 0.81; (2) using the RD index the classifier reached a balanced accuracy of 70% and a ROC-AUC of 0.80; (3) using permutation label testing with cross validation it was found that the classifier reached the above diagnostic performances not by chance (permutation-based *p* ≤ 0.05, for both indices). Interestingly, fornix was the most important structure for classification by both indices.

A set of WM structures was found to be important in the classification by trace and RD in our study, suggesting that LLD may be characterized by a widespread axonal injury (i.e., trace, RD) and/or demyelination (i.e., RD) in limbic (fornix, uncinate fasciculus, hypothalamus), frontopontine (internal capsule, cerebral peduncle), thalamo-cortical projection fibers (thalamus), fronto-striatal (caudate, external capsule), commissural fibers (corpus callosum), subcortical nuclei (substantia nigra, midbrain), brainstem and the cerebellum. In our study AdaBoost and Gboost outperformed SVM. This can be attributed to the data, the algorithms’ properties and modeling. Classification using all features together led also to a significant model with similar results to AdaBoost and GBoost, although feature importance were more scattered (Suppl. Fig. S6). This is not surprising as DTI indices are complementary in nature and the number of features is now dramatically increased (curse of dimensionality). Significant differences were also found between the groups in non-parametric statistical testing, but did not survive after multiple comparison correction, which can be attributed to factors such as the high number of tests performed and magnitude of the effects.

The literature on ML and DTI in LLD is limited^16,17^. Patel et al.^16^ used multimodal MRI data and the Alternating Decision Tree algorithm (an ensemble classifier, similar to AdaBoost) to classify 33 LLD and 35 non-depressed individuals and reported an accuracy of 87.3%. The authors suggest that global imaging measures (atrophy and global WM hyperintensity load) and non-imaging features (age and Mini-Mental Examination) are best predictors of diagnosis. In the study of Stolicyn et al.^17^ with 40 LLD cases and 40 controls using average FA and MD measures extracted for 19 bilateral and 5 unilateral tracts derived by TBSS and three classification models, the best classification accuracy achieved was 61.25% with MD features and the SVM classifier with optimized hyper parameters. Our study has focused on machine learning classification from an advanced DTI segmentation and the accuracy reached was 76% using both trace and RD indices (Table 2). Our results compare to accuracies reported in the recent review on ML classification in major depression using DWI measures, where they vary from 57 to 91.7%^18^.

Most of the studies in LLD with DTI in 3 Tesla have used voxel-based analyses (e.g., tract based spatial statistics-TBSS), Tractography, and ROI methods, and have mainly focused on differences between groups and in specific indices (i.e., FA and MD)^7,19^. Each of these methods carries drawbacks, such as operational burden, variability and error in manual ROI placement, fiber crossings in deterministic and complexity in probabilistic Tractography, as well as challenging investigation of the peripheral WM in voxel-based analysis. Furthermore, many predictions based on MRI variables have been made by univariate measures which reveal a moderate effect^20^. The segmentation framework used in our study allows high registration accuracy and accurate segmentations of the superficial WM, an area that is difficult to appreciate if population-averaged atlases are used^21^ as in voxel wise DTI analyses. In our analysis, we moved from a voxel-by-voxel type of analysis, where each of the hundreds of thousands of voxels is tested individually (lowering the statistical power) to a structure-by-structure one, with only 146 anatomically relevant imaging structures covering the whole brain WM and trained an ensemble classifier for diagnostic classification.

We found widespread diffusivity alterations within various anatomical structures as important for LLD diagnosis, and fornix was the most important structure. Based on MRI studies, many underlying circuits have been proposed to be pivotal in LLD, yet direct mechanistic links are missing. Our findings follow earlier studies. Specifically, limbic and frontal-subcortical circuitry disruption have been hypothesized in LLD^22,23^. Furthermore, brainstem nuclei have been involved in LLD^24^ and this is supported by pathological findings of neuronal loss in brainstem nuclei (e.g., raphe nucleus) and presence of Lewy bodies in subcortical nuclei (e.g., substantia nigra)^6,25^. Reduced FA and increased RD in the fronto-subcortical and limbic tracts (i.e., fornix and uncinate fasciculus) superior longitudinal fasciculus, and corpus callosum have been previously reported in LLD^26^. Another study found that MD was found to be increased in the fornix of patients with LLD compared to controls^27^. In a large sample from the UK Biobank Imaging Study, MD in anterior thalamic radiation, inferior fronto-occipital fasciculus, uncinate fasciculus, superior thalamic radiation, cingulate gyrus part of cingulum, and middle cerebellar peduncle has been associated with depressive symptoms in older individuals^28^. In an analysis on Alzheimer’s disease Neuroimaging Initiative data, the presence of subclinical depressive symptoms was associated with lower WM integrity mainly in the fornix, posterior cingulum, corpus callosum and inferior longitudinal fasciculus^29^. Another study showed that increased anatomical connectivity predominantly in a fronto-limbic network, defined by DTI probabilistic tractography predicted depression with 91.7% accuracy using SVM^30^. WM structures associated with subcortical gray matter nuclei (i.e., thalamus, caudate) insula and precuneus were found to be important in our study, which is in line with other studies. In particular, thalamic volume reductions were found to be significant in the meta-analysis of MRI studies in LLD^31^. Similarly, caudate nucleus^32,33^ and insula volume^34^ were found to be significantly lower in LLD. From a functional connectivity (FC) perspective, in the study of Lin et al.^15^ a diagnostic accuracy over 85% was achieved with the superior frontal gyrus, left insula, and right middle occipital gyrus using resting state (rs) fMRI and convolutional neural networks analysis. Increased right anterior insula-right dorsolateral prefrontal cortex rs-FC^35^, as well as altered fronto-cerebellar connectivity^36^ have been reported in older depressed adults with apathy. Another study found an increased FC of the left precuneus in patients with LLD compared to controls^37^.

Our study has the limitations of small sample and many independent variables and a main concern in this context is the risk of overfitting. We have taken actions to deal with this issue that are feasible for the data characteristics and first was the selection of the algorithm. AdaBoost combines a series of weak classifiers in order to build a more robust final classifier/prediction. It acts preventively to overfitting as it inherently performs a soft feature selection and iteratively adjusts the class prediction weights diversifying the data presented to the next cross validation iteration. By using stage wise additive modeling, AdaBoost slows down overfitting by optimizing certain parameters for the next iteration, while the rest from the previous iteration is held fixed (similar to a regularization procedure). The construction of simple base learners and the restricted use of 50 estimators, mitigates the influence of each individual learner, promotes efficient learning from imaging patterns in the data and prevents excessive learning from the training data (overfitting) resulting in a less biased model. This is further ensured by the use of stratified sampling to permit equal distribution of the classes in each cross validation fold. The use of k-fold cross validation creates models that have been tested on data unseen during the training. Even after all the above actions, a relative degree of overfitting cannot be excluded and future studies with larger samples will allow further investigation and accounting for this issue. It should be noted that the classifier has shown substantial improvement in the classification performance in atlas-based analyses^38^. Another limitation is that the model was not tested in an independent sample. To control this, we used cross validation testing the classifier on a subsample not used during the training; we also performed a permutation test to assess the statistical significance of the developed model. Evaluating our model given the sample characteristics is challenging. In this regard first we tried to control biases in the model (data normalization, stratified sampling Adaboost learning). We evaluated our model using k-fold cross validation and suitable performance measures along with their 95% Confidence Intervals. Importantly, we evaluate statistical significance using permutation testing. Additional classifiers and type of analysis were utilized to further investigate the feasibility of our study. We were able to create a valid model that performs consistently well across evaluation measures and within family of algorithms, and not by chance. The unbalanced data and differences in gender are limitations in our study. In this context we used robust methods for unbalanced data that permit a balanced representation of the two classes (stratified sampling) and combined with the classifier’s ability to focus on the misclassified cases allows effective capturing of the patterns and subtleties of the minority class improve the classifier’s ability to discriminate between unbalanced data. Regarding the gender differences, our model showed a small relationship between gender and DTI features and that it is not biased by gender (Suppl. Fig. S4). Another limitation is that the patients were medicated.

In conclusion, employing a multi-contrast, multi-atlas framework for DTI segmentation for the first time in LLD, to train and test the AdaBoost classifier, we suggest that trace and RD indices within structural networks involving the limbic, cortico-basal ganglia-thalamo-cortical loop, the brainstem, the external and internal capsules, corpus callosum and the cerebellum, are promising features in the diagnostic classification of LLD and HC subjects. The results need further validation and encourage the anatomical characterization of LLD using larger samples, as well as the combination of the adopted methods with other imaging, clinical, historic and environmental variables to develop stronger diagnostic models, evaluate interventions, and inform targeted treatments for a complex and heterogeneous mental disorder.

## Methods

### Participants

We recruited 26 consecutive patients from the Eginition hospital’s psychogeriatric unit. Inclusion criteria were age > 55 years, a DSM-IV-TR diagnosis of major depressive episode (single episode or recurrent) and no cognitive impairment, based on clinical criteria and a MMSE^39^ score ≥ 28. Depression was measured with the 15-item geriatric depression scale^40^. Exclusion criteria were presence of psychosis, suicidal ideation, a history of neurological/psychiatric condition (except depression), delirium, sensory deficits, alcohol/drug abuse, malignancy, and patients with MR incompatible implants and claustrophobia. All imaging data were reviewed by a neuroradiologist (GV) to identify unexpected lesions and by a medical physicist (EK) to identify participant or MRI-related artifacts. We also recruited using word of mouth 12 healthy controls (HC) matched for age, education and MMSE scores based on the same exclusion criteria.

### DTI and white matter segmentation

All participants underwent brain MRI in a 3 Tesla whole-body MRI scanner (Philips Achieva TX, Best, The Netherlands) equipped with an 8-channel head coil using the same imaging protocol. Imaging protocol included: (1) a high-resolution 3D axial T1-weigthed turbo field echo SENSE imaging (TE = 3.83 ms, TR = 8.31 ms. Flip angle = 8°. Field of view: 230 × 140 × 182 mm. In plane matrix size = 336 × 336 mm. A total of 200 slices with 0.7 mm thickness and no gaps covered the whole brain); ii) a T2 weighted dual turbo spin echo SENSE axial imaging (TE = 10.11 ms and 96 ms. TR = 3000 ms. Flip angle = 90°. Field of view: 240 × 144 × 210 mm. In plane matrix size = 256 × 256 mm. A total of 96 slices (2 × 48) with 3 mm thickness and no gaps covered the whole brain);and iii) for DTI imaging, a single-shot EPI sequence with SENSE parallel imaging (reduction factor 2.5). Imaging parameters were repetitiontime ≈ 7200 ms, echo time ≈ 74.5 ms, flip angle = 90°. The imaging volume for each subject included 60–70 axial slices of one b_min_ = 0 s/mm^2^ (b0) image, and 32 diffusion direction coding images with b_m_ = 700 s/mm^2^, acquired parallel to the anterior commissure/posterior commissure line, with 2.2 mm isotropic voxel size and image matrix 96 × 96, zero-filled to 256 × 256 and field of view 212 × 212 mm. DTI was repeated twice to improve the signal-to-noise ratio.

All DTI datasets were automatically post-processed and segmented using MRIcloud (www.mricloud.org)^41^ a valid^21,42^ and reproducible^43^ framework running on Windows. Briefly, the images are corrected for head motion and eddy-current-induced distortions^44^; image corruptions are automatically detected and rejected pixel-wise^45^. The two DTI sequences are then combined to estimate the tensor and derived maps using multivariate linear fitting. For the mapping, whole brain WM parcellation is performed employing a fully automated multi-contrast, multi-atlas segmentation and label fusion framework^46,47^. In the current implementation, a library of 8 atlases (“Adult_168labels_8atlases_V1”) of healthy individuals (mean age: 29 years) is used, along with a paired parcellation label map of 168 anatomical structures segmenting the whole brain (see Appendix 1 in the Supplemental Material). The segmentation workflow is graphically described in more detail in Supplemental Fig. S1.

### Image quantification and feature extraction

For the final image quantification, a threshold of FA > 0.2 was applied to remove the cortex while still preserving subject-specific anatomical features in these peripheral WM parts^21^. Of the 168 parcels originally segmenting the brain, 146 structures of interest were finally analyzed, in terms of FA volume (number of voxels with FA > 0.2), mean FA, diffusion trace (analogous to MD, as MD = trace/3), AD and RD. The ROI-Editor software was used for quantification^48^.

### Statistical analysis

Between group differences in DTI parcellation for each WM measure were examined using a non-parametric Mann–Whitney test in SPSS Statistics for Windows, version 28.0 (IBM Corp. Armonk, N.Y. USA).

### Machine learning analysis

**Figure 3:**
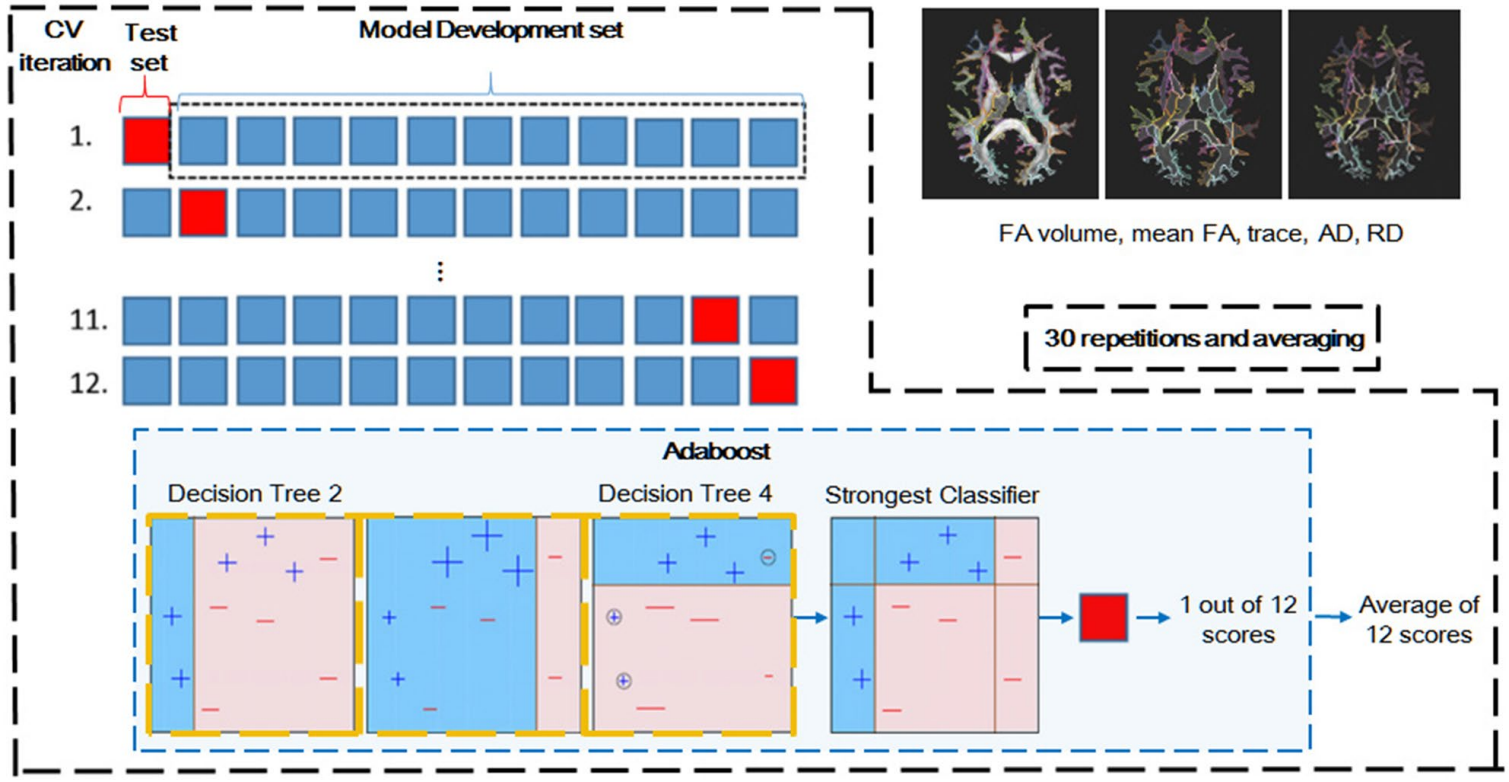
Illustration of the machine learning procedure followed in this study.

In a typical ΜL analysis, an algorithm is empirically learning through an iterative training-and-test procedure using the available data to accurately classify unseen data. For our data AdaBoost^49,50^ was used. Specifically, the SAMME.R (Stagewise Additive Modeling) algorithm^50^ was employed with default parameters (number of estimators = 50, learning rate = 1.0, max depth = 1) as implemented in Sci-kit learn. All classification analyses were performed in Python 3.6.13 (https://www.python.org), Scikit-learn 0.17.0 (http://scikit-learn.org/stable/)^51^. The classification procedures are illustrated in Fig. 3. Before training, the data were standardized by zeroing the mean of each attribute and scaling to unit variance using StandardScaler. Based on our sample characteristics, a stratified 12-fold cross validation was used, so that all data were used for training and validation (test), while maximizing the inclusion of HC in the training set (Fig. 3). In cross validation, the data are divided into k non-overlapping subsets (folds) of roughly equal size that serve as training and hold out/test sets. Then, boosting is applied on k-1 subsets while the left-out fold is used for validation and test. The process is repeated for each of the k subsets and a mean performance is obtained after repeating the entire process 30 times to account for bias in the initiation of the classifier and cross validation splitting (Fig. 3). Apart from Adaboost, we tested GBoost^52^ also from the ensemble boosting family, as well as support vector machines^53^. Gradient boosting or gradient boosted decision trees algorithm builds an additive model (i.e., the residuals of the previous fit round becomes the input for the next consecutive classifier, on which the trees are built) by combining multiple models moving in a step-by-step manner against the negative gradient to reduce the loss, in order to capture the maximum variance within the data and ultimately to create a strong predictive model based on regression trees. The pipeline for GBoost classification remained similar as that for AdaBoost. An implementation of libsvm^53^, was used for the classification with Support Vector classifier (SVC), as a supervised learning algorithm implemented in with Scikit-learn. After the data are projected in a high dimensional feature space, the classifier finds the plane (“hyperplane”) corresponding to a radial basis function kernel that best separates the two groups based on measurements (support vectors) closest to that plane. For SVM classification, feature selection was applied using the k = 60 best features with the highest F-scores between two random variables in univariate ANOVA. More details on the machine learning analysis can be found in the Supplemental Material.

Classification performance was evaluated in terms of mean accuracy and balanced accuracy, precision, recall (sensitivity), F1 score and ROC-AUC. Balanced accuracy is the arithmetic mean of sensitivity and specificity, as using accuracy only for model evaluation can bias towards overoptimistic results, especially with imbalanced datasets^54^. True positive rate (recall) and false positive rate are performance metrics useful for imbalanced class problems; ROC-AUC summarizes the trade-off between those two for every possible cut off, as the correlation between the class predicted by the classifier and the true class into which the case falls. ROC-AUC represents the power of the classifier measured in a scale that ranges from 0 (below chance performance) to 1 (perfectly accurate model) and 0.5 is random chance^55^. A combination of precision and recall is the F1-score.

$$ \begin{gathered} {\text{Accuracy}} = \frac{{{\text{TP}} + {\text{TN}}}}{{{\text{TP}} + {\text{TN}} + {\text{FP}} + {\text{FN}}}} \hfill \\ {\text{Balanced}}\;{\text{accuracy}} = \frac{{{\text{Sensitivity}} + {\text{Specificity}}}}{2} = \frac{{\frac{{{\text{TP}}}}{{{\text{TP}} + {\text{FN}}}} + \frac{{{\text{TN}}}}{{{\text{TN}} + {\text{FP}}}}}}{2} \hfill \\ {\text{Precision}} = \frac{{{\text{TP}}}}{{{\text{TP}} + {\text{FP}}}} \hfill \\ {\text{Recall}} = \frac{{{\text{TP}}}}{{{\text{TP}} + {\text{FN}}}} \hfill \\ {\text{F}}1{\text{-score}} = \frac{{2{\text{TP}}}}{{2{\text{TP}} + {\text{FP}} + {\text{FN}}}} \hfill \\ \end{gathered} $$

TP is the number of positive samples predicted as positive. FP is the number of negative samples predicted as positive. TN is the number of negative samples predicted as negative. FN is the number of positive samples predicted as negative.

### Statistical significance of the classifier

The classifier’s performance against chance was tested with a standard permutation procedure^56,57^ and ROC-AUC scores. This is a non-parametric approach in which the frequency distribution of a given performance metric (i.e., ROC-AUC) under the null hypothesis of independence is obtained, by randomly exchanging the labels (LLD or NC) associated with an instance. The entire training and test procedure is repeated multiple times using cross validation and an empirical P value is calculated by dividing the number of permutations resulted in a higher performance than that estimated with the actual sample by the number of permutations (i.e., 1000). If a significant association between the labels and WM features truly exists, then the average classification probability obtained after permutation is expected to be close to chance (i.e., around 50%). Permutation analysis was performed in Python.

### Ethics approval

The study was conducted according to the latest version of the Declaration of Helsinki and approved by the National and Kapodistrian University of Athens ethics committee (file number: 275/2016.05.31. ΑΔΑ: 6ΘΣ346Ψ8Ν2-ΒΣΡ). According to the permission for the MRI experiment: “Subjects have been informed by the doctor, with any detail about the diagnosis and the nature of his/her conditions, the kind and purpose of the medical intervention, and they gave their written consent for their participation in the brain imaging analysis with MRI. They give permission to the doctor and his assistants to make all the medical interventions they judge are necessary for their good health”. No other ethical permission is applied.

### Consent to participate

Written informed consent was obtained from all participants.

### Supplementary Information

Supplementary Information.

## Data Availability

All data are available from the corresponding author upon reasonable request.

## Code availability

The source code is available at https://github.com/exmath20005/DTI_ML_LLD_paper.

## References

[1] Allan CE, Ebmeier KB, Valkanova V (2014). Depression in older people is underdiagnosed. Practitioner.

[2] Byers AL, Yaffe K (2011). Depression and risk of developing dementia. Nat. Rev. Neurol..

[3] Robinson AC (2021). Mid to late-life scores of depression in the cognitively healthy are associated with cognitive status and Alzheimer's disease pathology at death. Int. J. Geriatr. Psychiatry.

[4] Smagula SF, Aizenstein HJ (2016). Brain structural connectivity in late-life major depressive disorder. Biol. Psychiatry Cognit. Neurosci. Neuroimag..

[5] Khundakar AA, Thomas AJ (2014). Cellular morphometry in late-life depression: A review of postmortem studies. Am. J. Geriatric Psychiatry.

[6] Tsopelas C (2011). Neuropathological correlates of late-life depression in older people. Br. J. Psychiatry.

[7] Wen MC, Steffens DC, Chen MK, Zainal NH (2014). Diffusion tensor imaging studies in late-life depression: Systematic review and meta-analysis. Int. J. Geriatr. Psychiatry.

[8] Wen J (2022). Characterizing heterogeneity in neuroimaging, cognition, clinical symptoms, and genetics among patients with late-life depression. JAMA Psychiat..

[9] Firbank MJ (2012). Relationship between progression of brain white matter changes and late-life depression: 3-year results from the LADIS study. Br. J. Psychiatry.

[10] Bezerra DM (2012). DTI voxelwise analysis did not differentiate older depressed patients from older subjects without depression. J. Psychiatr. Res..

[11] Choi KS (2014). Reconciling variable findings of white matter integrity in major depressive disorder. Neuropsychopharmacology.

[12] Jones EC, Liebel SW, Hallowell ES, Sweet LH (2019). Insula thickness asymmetry relates to risk of major depressive disorder in middle-aged to older adults. Psychiatry Res. Neuroimag..

[13] Shatte ABR, Hutchinson DM, Teague SJ (2019). Machine learning in mental health: a scoping review of methods and applications. Psychol. Med..

[14] Zhang, L. *et al*. Hybrid representation learning for cognitive diagnosis in late-life depression over 5 years with structural MRI. 10.48550/arxiv.2212.12810 (2022). 10.1016/j.media.2024.103135 PMC11016377 38461654

[15] Lin C (2023). Automatic diagnosis of late-life depression by 3D convolutional neural networks and cross-sample Entropy analysis from resting-state fMRI. Brain Imaging Behav..

[16] Patel, M. *et al*. Machine learning approaches for integrating clinical and imaging features in late-life depression classification and response prediction. *Int. J. Geriatric Psychiatry*. **30**(10), 1056–1067 (2015). 10.1002/gps.4262 PMC4683603 25689482

[17] Stolicyn A (2020). Automated classification of depression from structural brain measures across two independent community-based cohorts. Hum. Brain Mapp..

[18] Gao S, Calhoun VD, Sui J (2018). Machine learning in major depression: From classification to treatment outcome prediction. CNS Neurosci. Ther..

[19] Rashidi-Ranjbar N, Miranda D, Butters MA, Mulsant BH, Voineskos AN (2020). Evidence for structural and functional alterations of frontal-executive and corticolimbic circuits in late-life depression and relationship to mild cognitive impairment and dementia: A systematic review. Front. Neurosci..

[20] Winter NR (2022). Quantifying deviations of brain structure and function in major depressive disorder across neuroimaging modalities. JAMA Psychiat..

[21] Oishi K (2009). Atlas-based whole brain white matter analysis using large deformation diffeomorphic metric mapping: Application to normal elderly and Alzheimer's disease participants. Neuroimage.

[22] Alexopoulos GS (2002). Frontostriatal and limbic dysfunction in late-life depression. Am. J. Geriatr. Psychiatry.

[23] Phillips ML, Drevets WC, Rauch SL, Lane RD (2003). Neurobiology of emotion perception II: Implications for major psychiatric disorders. Biol. Psychiatry.

[24] Smith GS (2021). Positron emission tomography imaging of serotonin degeneration and beta-amyloid deposition in late-life depression evaluated with multi-modal partial least squares. Transl. Psychiatry.

[25] Wilson R (2016). Late-life depression is not associated with dementia-related pathology. Neuropsychology (Journal).

[26] Sexton CE (2012). Magnetic resonance imaging in late-life depression: Multimodal examination of network disruption. Arch. General Psychiatry.

[27] Li W (2014). Effects of the coexistence of late-life depression and mild cognitive impairment on white matter microstructure. J. Neurol. Sci..

[28] Shen X (2019). White matter microstructure and its relation to longitudinal measures of depressive symptoms in mid- and late life. Biol. Psychiatry.

[29] Touron E (2022). Depressive symptoms in cognitively unimpaired older adults are associated with lower structural and functional integrity in a frontolimbic network. Mol. Psychiatry.

[30] Fang P (2012). Increased cortical-limbic anatomical network connectivity in major depression revealed by diffusion tensor imaging. PLOS ONE.

[31] Sexton CE, Mackay CE, Ebmeier KP (2013). A systematic review and meta-analysis of magnetic resonance imaging studies in late-life depression. Am. J. Geriatric Psychiatry.

[32] Butters MA (2009). Three-dimensional surface mapping of the caudate nucleus in late-life depression. Am. J. Geriatric Psychiatry.

[33] Kumar A (2004). Biophysical changes in normal-appearing white matter and subcortical nuclei in late-life major depression detected using magnetization transfer. Psychiatry Res. Neuroimaging.

[34] Laird KT (2019). Anxiety symptoms are associated with smaller insular and orbitofrontal cortex volumes in late-life depression. J. Affect. Disord..

[35] Yuen GS (2014). The salience network in the apathy of late-life depression. Int. J. Geriatr. Psychiatry.

[36] Alalade E, Denny K, Potter GG, Steffens DC, Wang LV (2011). Altered cerebellar-cerebral functional connectivity in geriatric depression. PLOS ONE.

[37] Alexopoulos GS (2013). Functional connectivity in apathy of late-life depression: A preliminary study. J. Affect. Disord..

[38] Zang J (2021). Effects of brain atlases and machine learning methods on the discrimination of schizophrenia patients: A multimodal MRI study. Front. Neurosci..

[39] Folstein MF, Folstein SE, McHugh PR (1975). "Mini-mental state". A practical method for grading the cognitive state of patients for the clinician. J. Psychiatric Res..

[40] Fountoulakis KN (1999). The validation of the short form of the Geriatric Depression Scale (GDS) in Greece. Aging (Milan, Italy).

[41] Mori S (2016). MRICloud: Delivering high-throughput MRI neuroinformatics as cloud-based software as a service. Comput. Sci. Eng..

[42] Ceritoglu C (2009). Multi-contrast large deformation diffeomorphic metric mapping for diffusion tensor imaging. NeuroImage.

[43] Rezende TJR (2019). Test-retest reproducibility of a multi-atlas automated segmentation tool on multimodality brain MRI. Brain Behav..

[44] Penny WD, Friston KJ, Ashburner JT, Kiebel SJ, Nichols TE (2011). Statistical Parametric Mapping: The Analysis of Functional Brain Images.

[45] Li Y (2013). Image corruption detection in diffusion tensor imaging for post-processing and real-time monitoring. PLOS ONE.

[46] Tang X (2014). Multi-contrast multi-atlas Parcellation of diffusion tensor imaging of the human brain. PLOS ONE.

[47] Wang H (2013). Multi-atlas segmentation with joint label fusion. IEEE Trans. Pattern Anal. Mach. Intell..

[48] van Jiang H, Zijl PC, Kim J, Pearlson GD, Mori S (2006). DtiStudio: Resource program for diffusion tensor computation and fiber bundle tracking. Computer Methods Programs Biomed..

[49] Freund, Y. & Schapire, R. E. A desicion-theoretic generalization of on-line learning and an application to boosting. *SpringerLink* (1995). 10.1007/3-540-59119-2_166.

[50] Zhu J, Zou H, Rosset S, Hastie T (2009). Multi-class AdaBoost. Stat. Interface..

[51] Pedregosa F (2011). Scikit-learn: Machine learning in Python. J. Mach. Learn. Res..

[52] Friedman, J. H. Greedy function approximation: A gradient boosting machine. *Ann. Stat*. **29**, 1189–1232 (2001).

[53] Chang C-C, Lin C-J (2011). LIBSVM. ACM Trans. Intell. Syst. Technol..

[54] Galar M, Fernández AÁ, Barrenechea E, Bustince H, Herrera F (2012). A review on ensembles for the class imbalance problem: bagging-, boosting-, and hybrid-based approaches. IEEE Trans. Syst. Man Cybernet..

[55] Fawcett T (2006). An introduction to ROC analysis. Pattern Recognit. Lett..

[56] Cui Z, Xia Z, Su M, Shu H, Gong G (2016). Disrupted white matter connectivity underlying developmental dyslexia: A machine learning approach. Hum. Brain Mapp..

[57] Good PI (2000). Permutation Tests: A Practical Guide to Resampling Methods for Testing Hypotheses.

