# Supplementary material for: Brain multi-contrast, multi-atlas segmentation of diffusion tensor imaging and ensemble learning automatically diagnose late-life depression
Source: Sci Rep. 2023 Dec 20;13:22743. doi: 10.1038/s41598-023-49935-z (PMC10733280; doi:10.1038/s41598-023-49935-z)
Supplement: Supplementary file 1 — Supplementary Information. [file 41598_2023_49935_MOESM1_ESM.pdf]

Title: **Brain multi-contrast, multi-atlas segmentation of diffusion tensor imaging and ensemble learning automatically diagnose late-life depression**

Kostas Siarkos[1*], Efstratios Karavassilis[2,3], Georgios Velonakis[3], Charalabos Papageorgiou[4], Nikolaos Smyrnis[5], Nikolaos Kelekis[3], Antonios Politis[1,6]

[1]Division of Geriatric Psychiatry, First Department of Psychiatry, National and Kapodistrian University of Athens, Athens, Greece.
[2]Medical School, Democritus University of Thrace, Alexandroupolis, Greece
[3]Second Department of Radiology, Attikon General University Hospital, School of Medicine, National and Kapodistrian University of Athens, Athens, Greece
[4]First Department of Psychiatry, School of Medicine, Eginition Hospital, National and Kapodistrian University of Athens, Athens, Greece
[5]Second Department of Psychiatry, Attikon General University Hospital, School of Medicine, National and Kapodistrian University of Athens, Athens, Greece
[6]Department of Psychiatry, Division of Geriatric Psychiatry and Neuropsychiatry, Johns Hopkins Medical School, Baltimore, USA

[*]**Corresponding author:** Address: 72-74 Vasilissis Sofias Ave. Athens 11528, Greece. E-mail: ksiarkos@med.uoa.gr. Tel: +306974419811. ORCID: 0000-0002-3366-2989.

Supplementary Material

**Regions**	**Raw *P*-values**
**FA Volume**	
fornix-stria terminalis R	p=0.005
retro lenticular internal capsule L	p=0.005
inferior cerebellar peduncle L	p=0.011
cerebral peduncle L	p=0.012
corticospinal tract R	p=0.021
retro-lenticular internal capsule R	p=0.021
cerebellum branch A L	p=0.023
superior temporal gyrus R gm	p=0.025
corticospinal tract L	p=0.03
sagittal stratum R	p=0.03
cuneus R gm	p=0.03
cingulum R	p=0.033
posterior corona radiata L	p=0.033
cingulate gyrus L gm	p=0.045
inferior frontal gyrus L gm	p=0.045
reticular formation R	p=0.049
**FA**	
medulla L	p=0.011
midbrain L	p=0.042

**Trace**	
lingual gyrus R gm	p=0.028
cerebellum	p=0.03
medulla L	p=0.042
**AD**	
cerebellum	p=0.03
**RD**	
cerebellum	p=0.023
medulla L	p=0.033

Supplementary Table S1 Regions with statistically significant differences between the groups in Mann-Whitney test and their significance level listed in descending order
Gm, grey matter; L, left; R, right.

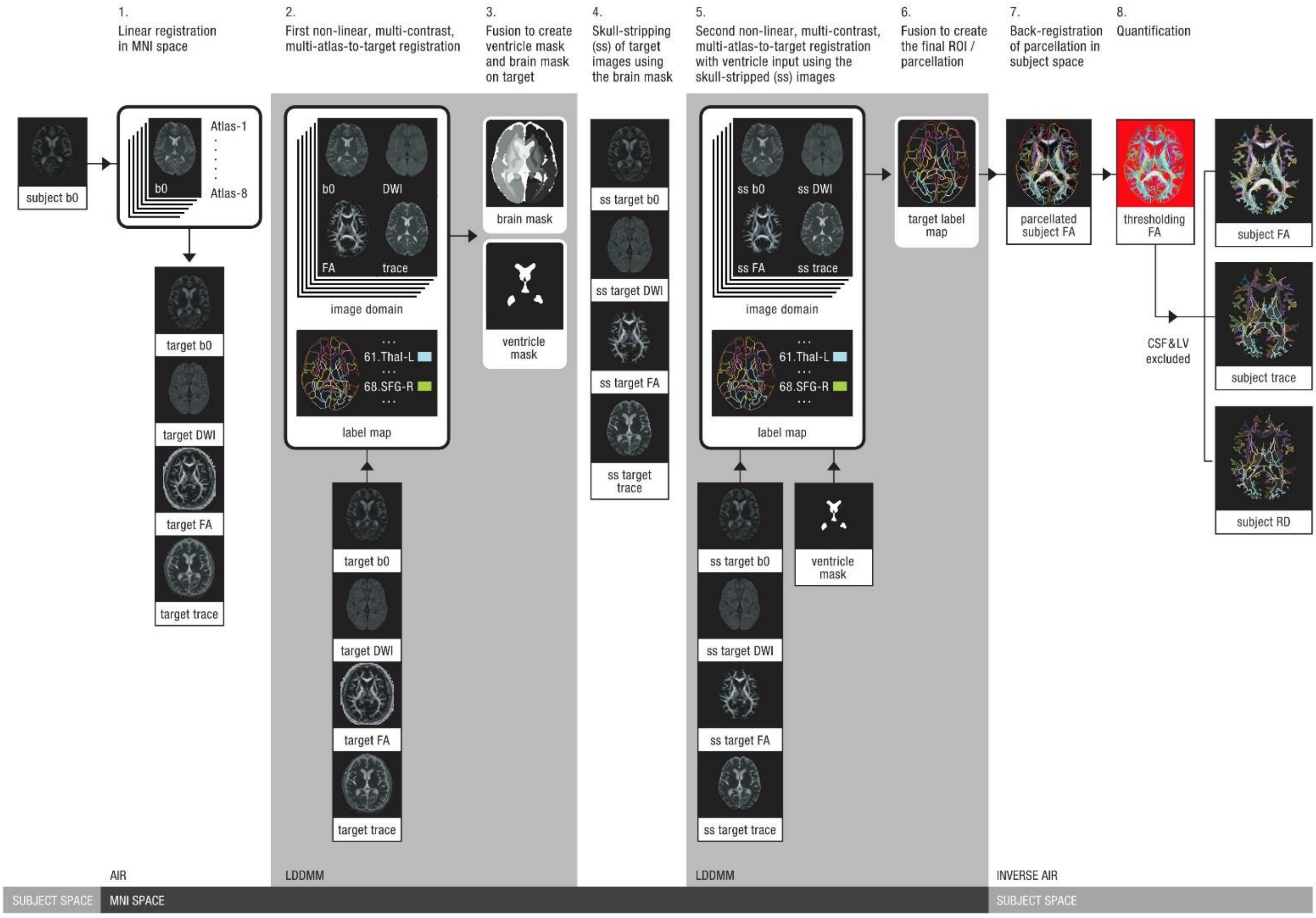

Supplementary Fig. S1 Schematic representation of the automatic image segmentation pipeline with a description for each step (1 to 8). Note: The "subject AD" map output, also generated at step 8, is not shown. AIR: Automated Image Registration. LDDMM: Large Deformation Diffeomorphic Metric Mapping

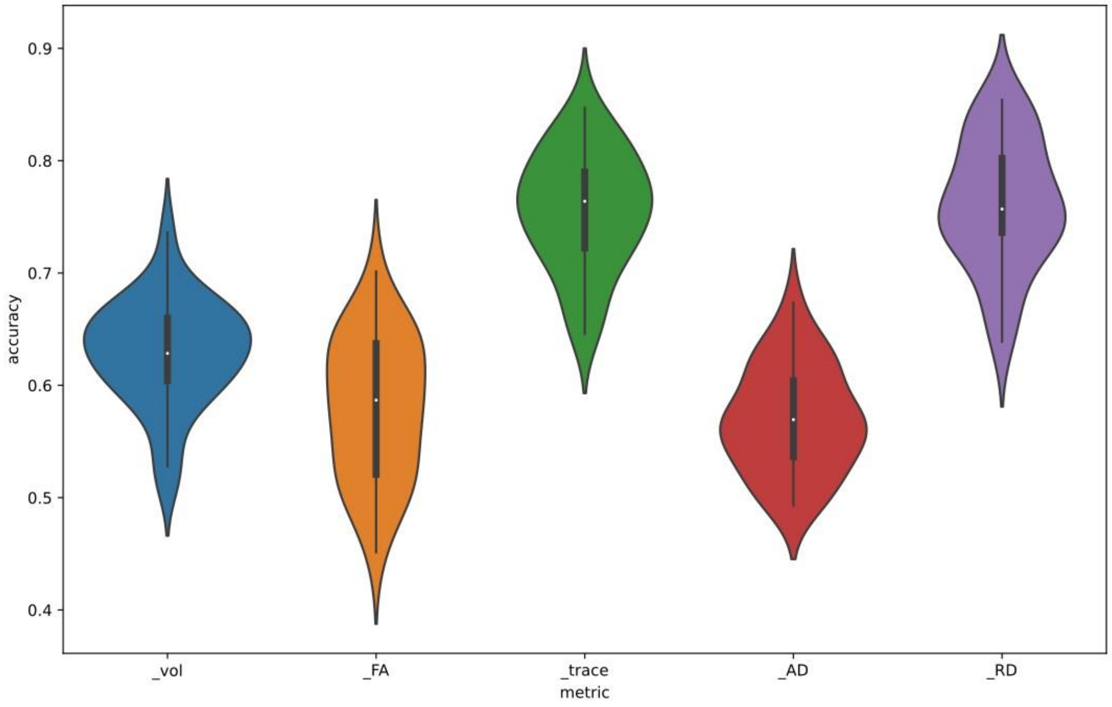
accuracy
0.4
0.5
0.6
0.7
0.8
0.9
_vol
_FA
_trace
_AD
_RD
metric

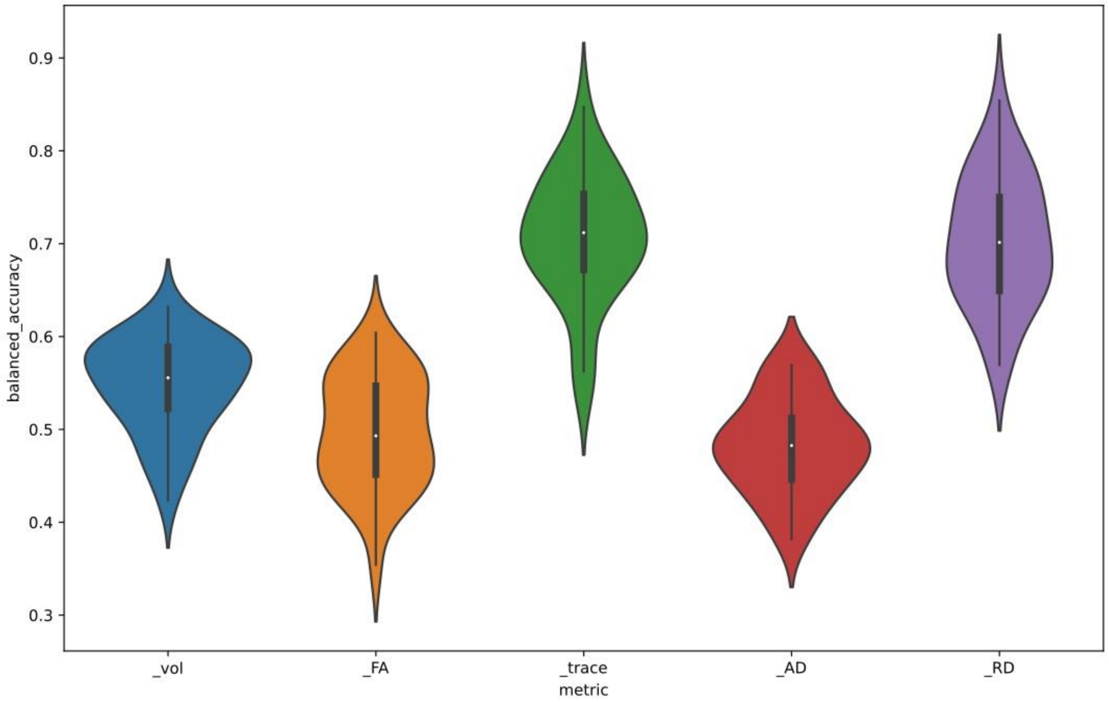
balanced_accuracy
0.3
0.4
0.5
0.6
0.7
0.8
0.9
_vol
_FA
_trace
_AD
_RD
metric

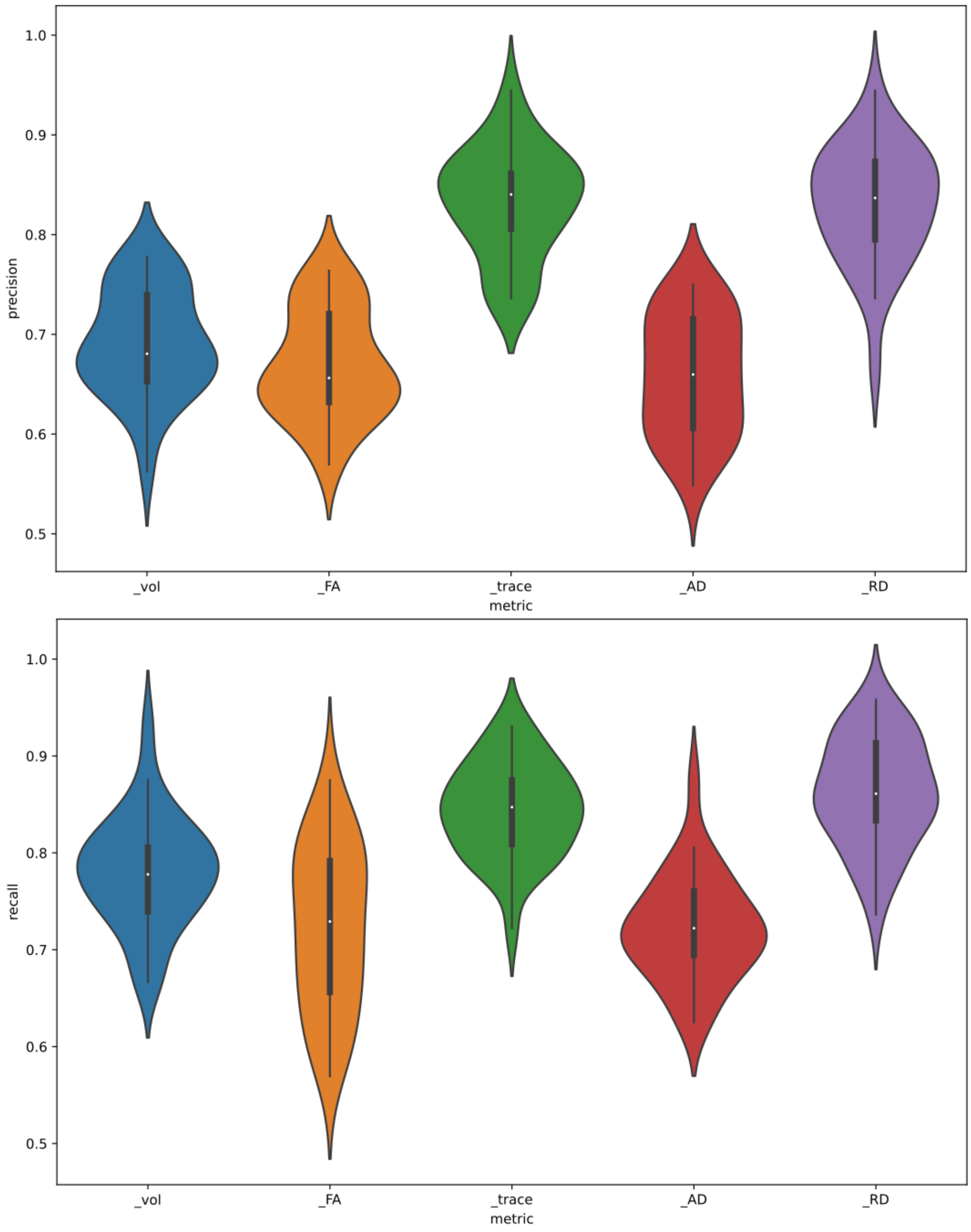
precision
1.0
0.9
0.8
0.7
0.6
0.5
_vol
_FA
_trace
_AD
_RD
metric
recall
1.0
0.9
0.8
0.7
0.6
0.5
_vol
_FA
_trace
_AD
_RD
metric

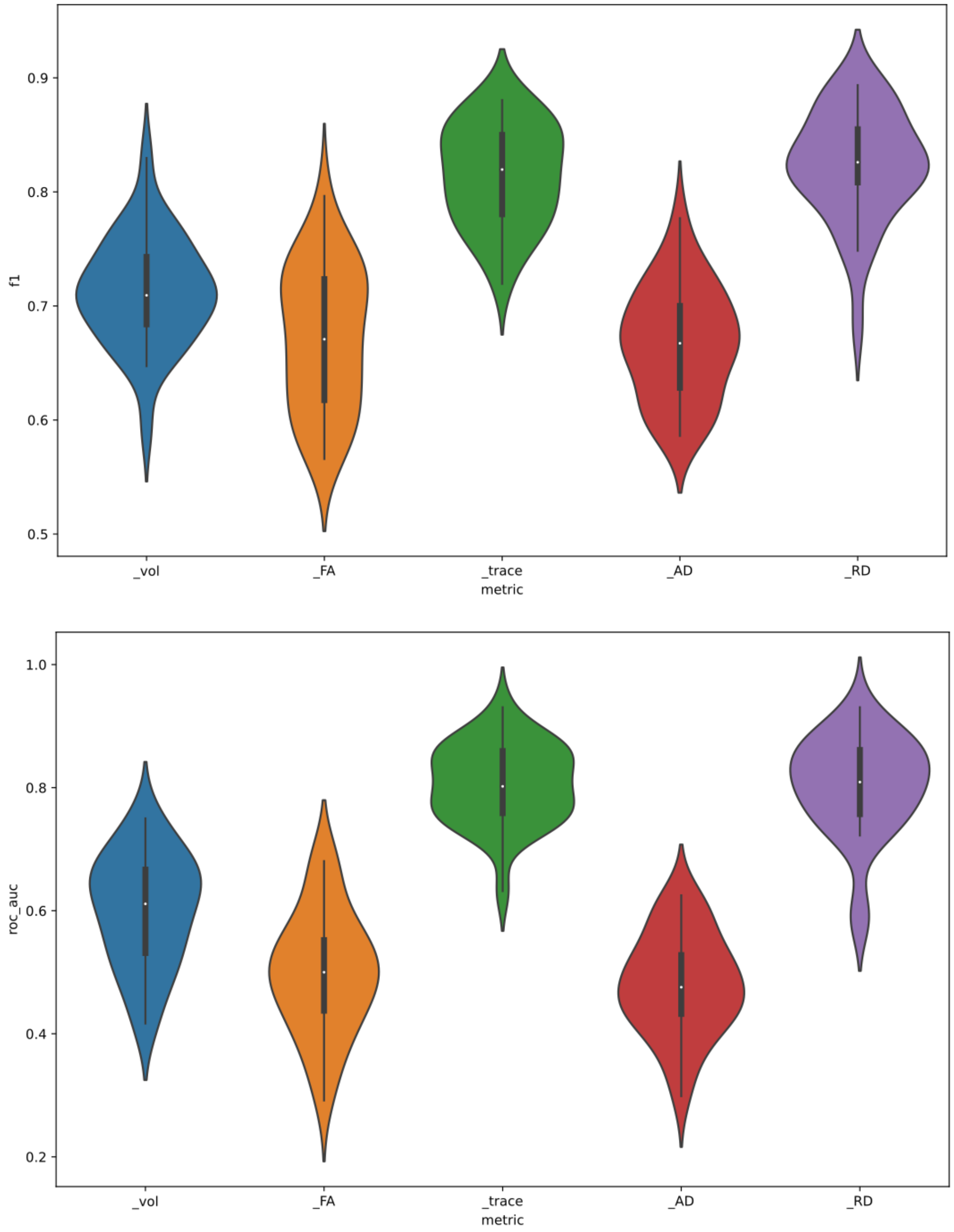

Supplementary Fig. S2 Violin plots of the classification performances for each evaluation metric. The colored component (density plot) represents the frequency of each performance score (denser=more frequent). The black component inside the density plot is actually a boxplot showing the 95% Confidence Interval (vertical thin bar) of the range of values within which the true performance is likely to fall, their interquartile range (vertical bold bar) and median (white point inside the bold bar).

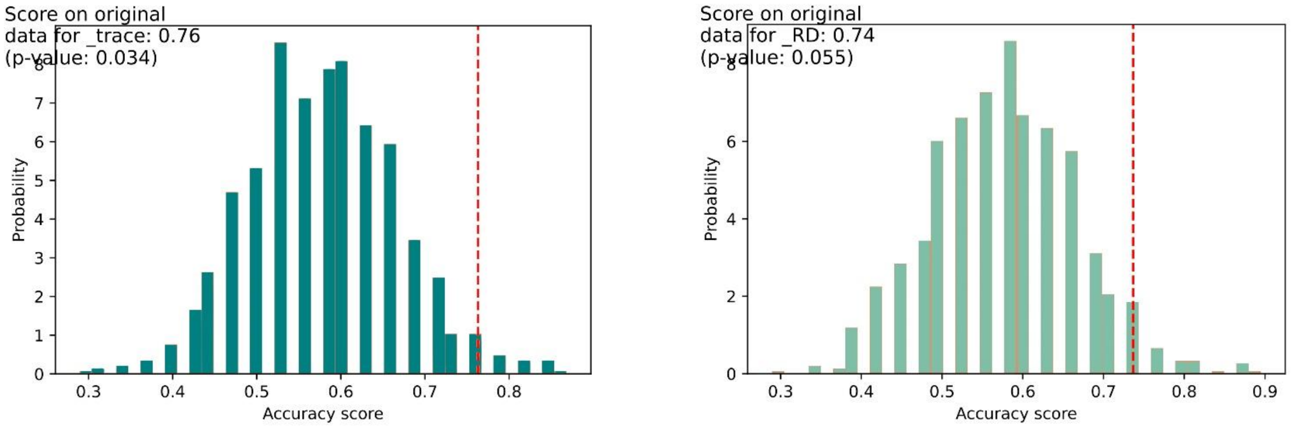

Supplementary Fig. S3 Distribution of balanced accuracy scores (shown in green) obtained with the AdaBoost classifier using cross validation after 1000 label permutations, compared with the score obtained with the actual data (dashed red line) for trace (left) and RD (right). Differences are considered significant at the 95% confidence interval level.

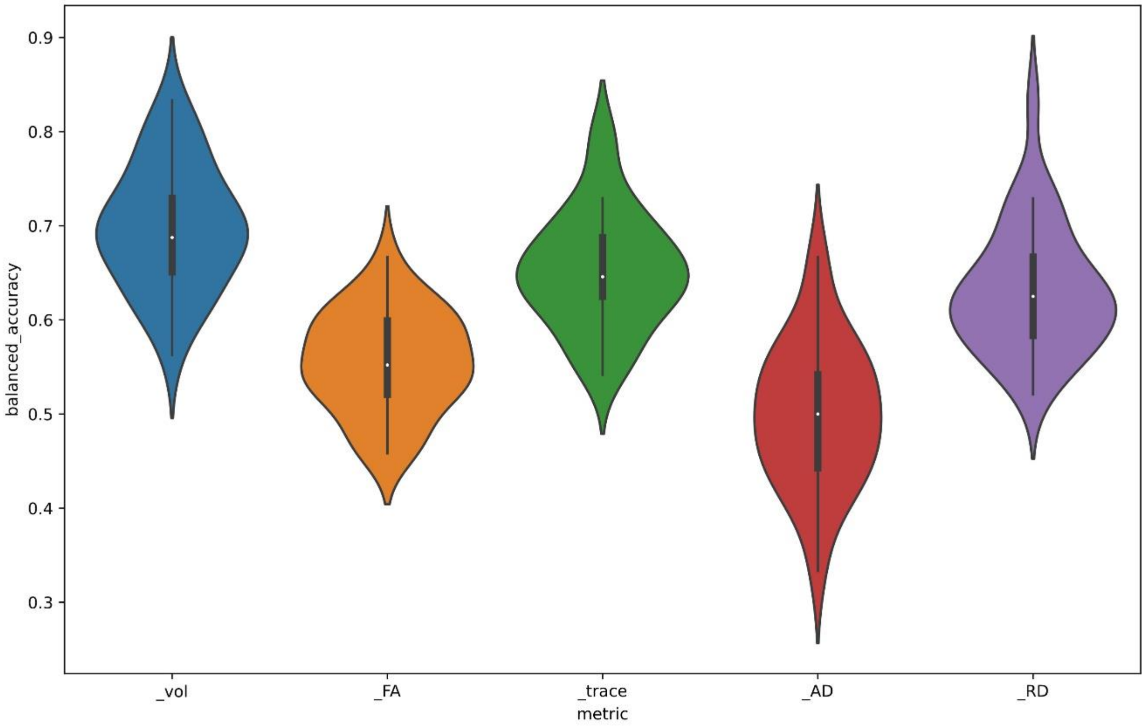

Supplementary Fig. S4 Violin plots for balanced accuracy of DTI indices to classify the gender. It can be seen that gender is poorly predicted by the DTI indices.

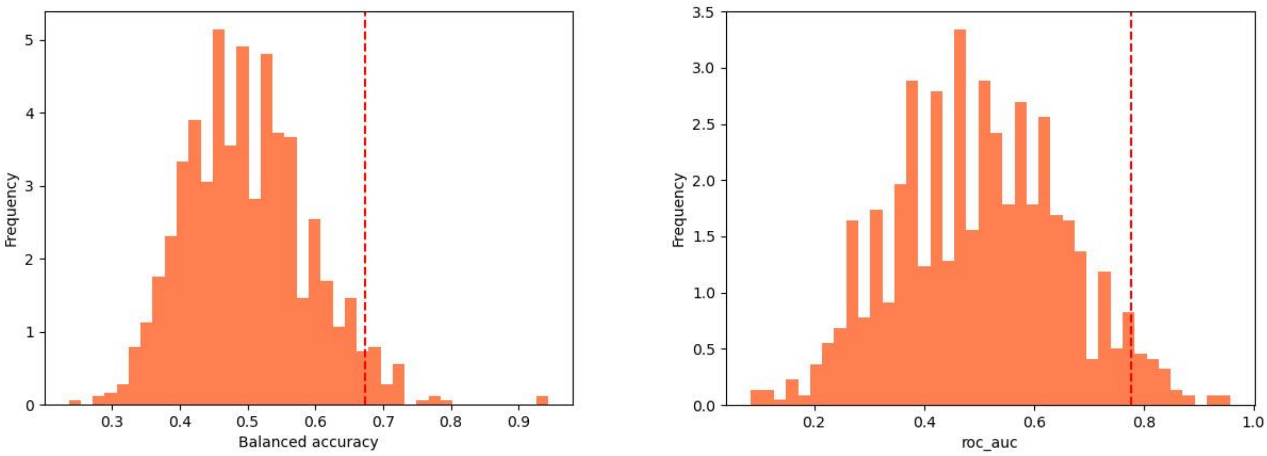

Supplementary Fig. S5 Distribution of balanced accuracy scores and ROC_AUC scores obtained with the AdaBoost classifier and all features using cross validation after 1000 label permutations, compared with the score obtained with the actual data (dashed red line). Differences are considered significant at the 95% confidence interval level.

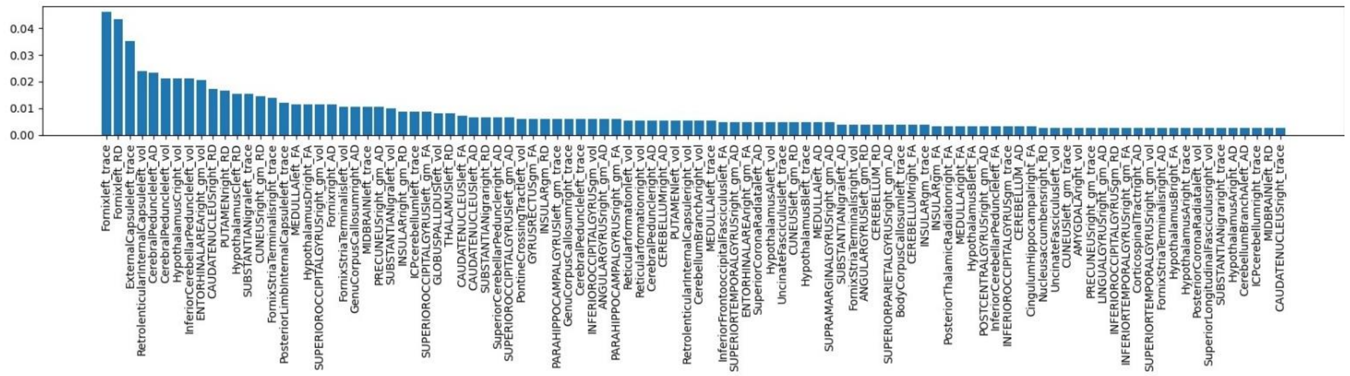

Supplementary Fig. S6 Feature importances with AdaBoost classification and all features as input.

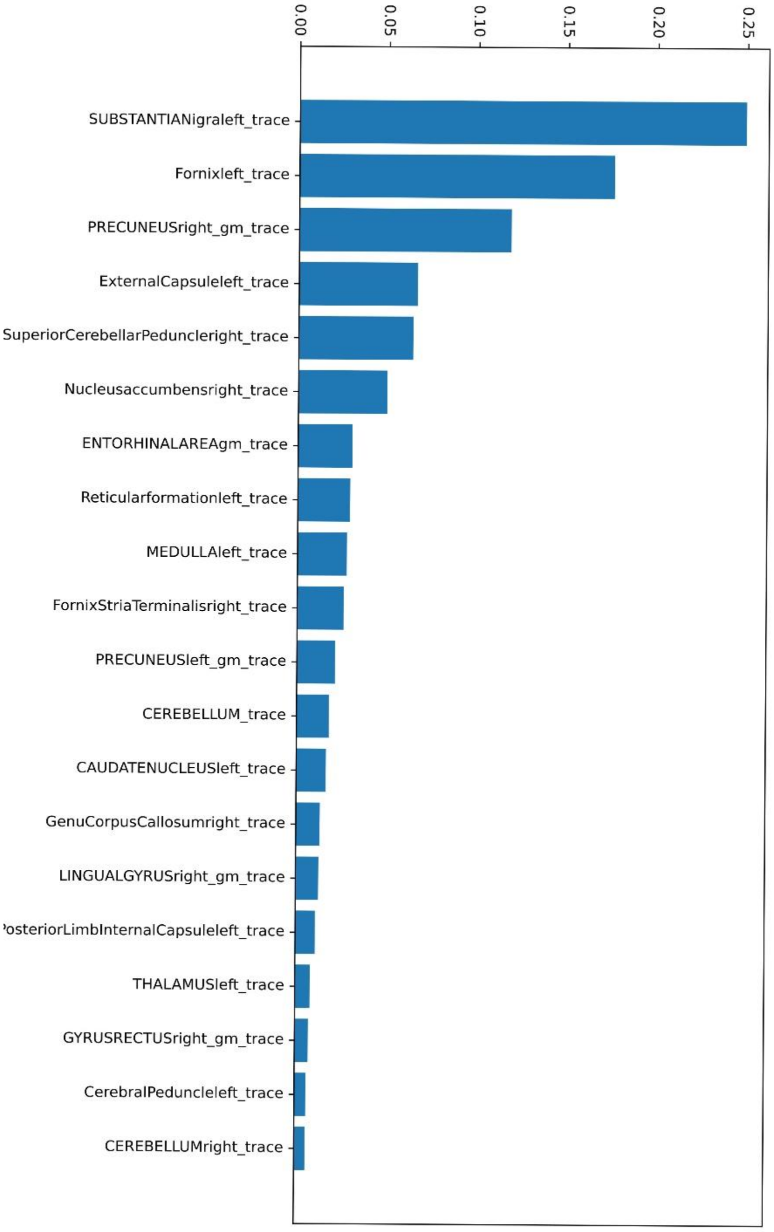

Supplementary Fig. S7 Trace feature importances in GBoost classification.

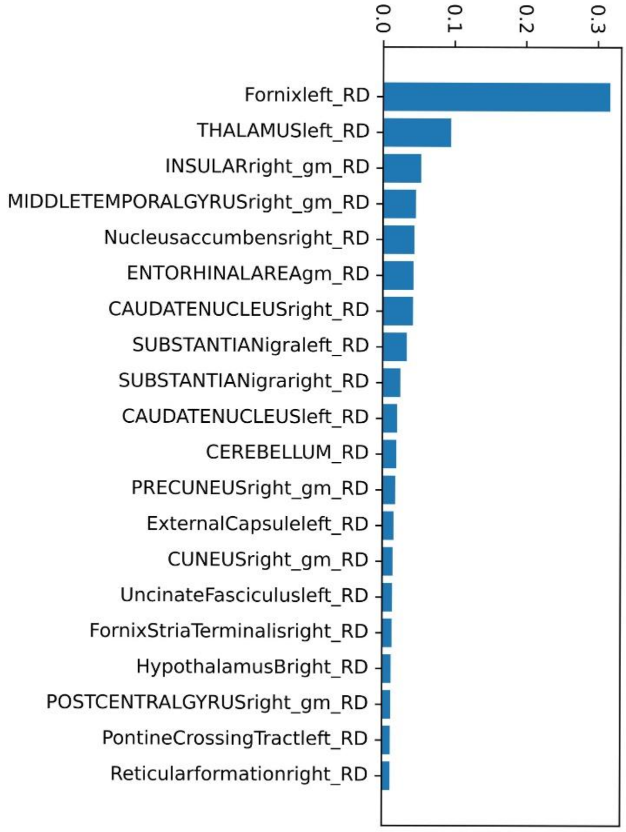

Supplementary Fig. S8 RD feature importances in GBoost classification.

Structure#	Structure Label
1	SUPERIOR PARIETAL GYRUS left (gm)
2	CINGULATE GYRUS left (gm)
3	SUPERIOR FRONTAL GYRUS left (gm)
4	MIDDLE FRONTAL GYRUS left (gm)
5	INFERIOR FRONTAL GYRUS left (gm)
6	PRECENTRAL GYRUS left (gm)
7	POSTCENTRAL GYRUS left (gm)
8	ANGULAR GYRUS left (gm)
9	PRE-CUNEUS left (gm)
10	CUNEUS left (gm)
11	LINGUAL GYRUS left (gm)
12	FUSIFORM GYRUS left (gm)
13	PARAHIPPOCAMPAL GYRUS left (gm)
14	SUPERIOR OCCIPITAL GYRUS left (gm)
15	INFERIOR OCCIPITAL GYRUS left (gm)
16	MIDDLE OCCIPITAL GYRUS left (gm)

17	ENTORHINAL AREA left (gm)
18	SUPERIOR TEMPORAL GYRUS left (gm)
19	INFERIOR TEMPORAL GYRUS left (gm)
20	MIDDLE TEMPORAL GYRUS left (gm)
21	LATERAL FRONTO-ORBITAL GYRUS left (gm)
22	MIDDLE FRONTO-ORBITAL GYRUS left (gm)
23	SUPRAMARGINAL GYRUS left (gm)
24	GYRUS RECTUS left (gm)
25	INSULAR left (gm)
26	AMYGDALA left
27	HIPPOCAMPUS left
28	CEREBELLUM left
29	Corticospinal Tract left
30	Inferior Cerebellar Peduncle left
31	Medial Lemniscus left
32	Superior Cerebellar Peduncle left
33	Cerebral Peduncle left
34	Anterior Limb of Internal Capsule left
35	Posterior Limb of Internal Capsule left
36	Posterior Thalamic Radiation left
37	Anterior Corona Radiata left

38	Superior Corona Radiata left
39	Posterior Corona Radiata left
40	Cingulum left
41	Cingulum Hippocampal left
42	Fornix Stria Terminalis left
43	Superior Longitudinal Fasciculus left
44	Superior Fronto-occipital Fasciculus left
45	InferiorFronto-occipitalFasciculus left
46	SagittalStratum left
47	ExternalCapsule left
48	UncinateFasciculus left
49	PontineCrossingTract left
50	MiddleCerebellarPeduncle left
51	Fornix left
52	Genu of Corpus Callosum left
53	Body of Corpus Callosum left
54	Splenium of Corpus Callosum left
55	Retrolenticular Internal Capsule left
56	Red Nucleus left
57	Substantia Nigra left
58	Tapetum left

59	CAUDATE NUCLEUS left
60	PUTAMEN left
61	THALAMUS left
62	GLOBUS PALLIDUS left
63	MIDBRAIN left
64	Reticular formation left
65	MEDULLA left
66	SUPERIOR PARIETAL GYRUS right (gm)
67	CINGULATE GYRUS right (gm)
68	SUPERIOR FRONTAL GYRUS right (gm)
69	MIDDLE FRONTAL GYRUS right (gm)
70	INFERIOR FRONTAL GYRUS right (gm)
71	PRECENTRAL GYRUS right (gm)
72	POSTCENTRAL GYRUS right (gm)
73	ANGULAR GYRUS right (gm)
74	PRECUNEUS right (gm)
75	CUNEUS right (gm)
76	LINGUAL GYRUS right (gm)
77	FUSIFORM GYRUS right (gm)
78	PARAHIPPOCAMPAL GYRUS right (gm)
79	SUPERIOR OCCIPITAL GYRUS right (gm)

80	INFERIOR OCCIPITAL GYRUS right (gm)
81	MIDDLE OCCIPITAL GYRUS right (gm)
82	ENTORHINAL AREA right (gm)
83	SUPERIOR TEMPORAL GYRUS right (gm)
84	INFERIOR TEMPORAL GYRUS right (gm)
85	MIDDLE TEMPORAL GYRUS right (gm)
86	LATERAL FRONTO-ORBITAL GYRUS right (gm)
87	MIDDLE FRONTO-ORBITAL GYRUS right (gm)
88	SUPRAMARGINAL GYRUS right (gm)
89	GYRUS RECTUS right (gm)
90	INSULAR right (gm)
91	AMYGDALA right
92	HIPPOCAMPUS right
93	CEREBELLUM right
94	Corticospinal Tract right
95	Inferior Cerebellar Peduncle right
96	Medial Lemniscus right
97	Superior Cerebellar Peduncle right
98	Cerebral Peduncle right
99	Anterior Limb of Internal Capsule right
100	Posterior Limb of Internal Capsule right

101	Posterior Thalamic Radiation right
102	Anterior Corona Radiata right
103	Superior Corona Radiata right
104	Posterior Corona Radiata right
105	Cingulum right
106	Cingulum Hippocampal right
107	Fornix Stria Terminalis right
108	Superior Longitudinal Fasciculus right
109	Superior Fronto-occipital Fasciculus right
110	Inferior Fronto-occipital Fasciculus right
111	Sagittal Stratum right
112	External Capsule right
113	Uncinate Fasciculus right
114	Pontine Crossing Tract right
115	Middle Cerebellar Peduncle right
116	Fornix right
117	Genu of Corpus Callosum right
118	Body of Corpus Callosum right
119	Splenium of Corpus Callosum right
120	Retrolenticular Internal Capsule right
121	Red Nucleus right

122	Substantia Nigra right
123	Tapetum right
124	CAUDATE NUCLEUS right
125	PUTAMEN right
126	THALAMUS right
127	GLOBUS PALLIDUS right
128	MIDBRAIN right
129	Reticular formation right
130	MEDULLA right
131	Nucleus accumbens left
132	Nucleus accumbens right
133	Hypothalamus A left
134	Hypothalamus A right
135	OpticTract left
136	OpticTract right
137	Hypothalamus B left
138	Hypothalamus B right
139	Mammillary body right
140	Mammillary body left
141	Hypothalamus C left
142	Hypothalamus C right

143	Hypothalamus D left
144	Hypothalamus D right
145	Hypothalamus E left
146	Hypothalamus E right
147	Hypothalamus F left
148	Hypothalamus F right
149	LVL_frontal left
150	LVL_frontal right
151	LVL_body left
152	LVL_body right
153	LVL_atrium left
154	LVL_atrium right
155	LVL_occipital left
156	LVL_occipital right
157	LVL_temporal left
158	LVL_temporal right
159	III and IV ventricle
160	Superior Longitudinal Fasciculus-t left
161	Superior Longitudinal Fasciculus-t right
162	ICP-cerebellum left
163	ICP-cerebellum right

164	Cerebellum Branch-A left
165	Cerebellum Branch-A right
166	Cerebellum Branch-B left
167	Cerebellum Branch-B right
168	CSF

Appendix 1. All WM structures defined in the original parcellation maps currently featured in MRIcloud and used in the present study
